# Editorial: The role of soy in human health and disease

**DOI:** 10.3389/fnut.2023.1278094

**Published:** 2023-09-15

**Authors:** Mauro Lombardo, Alessandra Feraco, Gianluca Rizzo

**Affiliations:** ^1^Department of Human Sciences and Promotion of the Quality of Life, San Raffaele Open University, Rome, Italy; ^2^Laboratory of Cardiovascular Endocrinology, San Raffaele Research Institute, Istituti di Ricovero e Cura a Carattere Scientifico (IRCCS) San Raffaele Roma, Rome, Italy; ^3^Independent Researcher, Via Venezuela, Messina, Italy

**Keywords:** soybean, nutrition, health benefits, environmental impact, cancer, diabetes

In recent years, the relationship between dietary choices and their influence on various aspects of health has garnered significant attention (1, 2). Particularly, the effects of soy consumption (3) and plant-based diets (4), have become an area of interest for researchers and healthcare professionals.

Soy, a versatile legume, has gained significant attention due to its potential health benefits (5–7). In the manuscript “*Inverse association of daily fermented soybean paste ('jang') intake with metabolic syndrome risk, especially body fat and hypertension, in men of a large hospital-based cohort*,” Jeong et al. present findings that suggest a potential link between fermented soybean paste consumption and a reduced risk of metabolic syndrome. This study sheds light on the intricate relationship between soy-based dietary components and metabolic health.

In “*The health effects of soy: a reference guide for health professionals*” Messina et al. provide a comprehensive reference guide that aids health professionals in understanding the various health implications of soy consumption. This resource serves as a valuable tool for practitioners seeking evidence-based insights into the benefits and potential concerns associated with incorporating soy into the diet.

Addressing global food security challenges, Messina discusses the role of soybeans in meeting the caloric and protein needs of a growing population. This perspective, titled “*Soybeans can help address the caloric and protein needs of a growing global population*,” underscores the significance of soy as a sustainable and nutritious food source for a burgeoning world population.

The potential link between soy consumption and cancer risk is explored in the systematic review by Fan et al. titled “*Intake of soy, soy isoflavones and soy protein and risk of cancer incidence and mortality*”. This study delves into the nuanced relationship between soy components and cancer outcomes, providing valuable insights for public health considerations.

As the field of nutritional science advances, these findings are instrumental in guiding evidence-based dietary recommendations and health interventions. With the growing interest in personalized nutrition, the diverse effects of soy on various health facets provide a basis for tailoring dietary strategies to individual needs (8) (Messina et al.). Moreover, the multifunctional properties of soy-derived bioactive compounds call for further investigations into their molecular mechanisms, potentially uncovering novel therapeutic avenues (9).

To further explore the intricate relationship between soy and human health, it is crucial to consider the interaction with environmental issues. As global attention to sustainability grows, understanding the link between soy consumption and environmental issues opens up new directions for research. Sustainable agricultural practices, supply chain dynamics and the environmental impact of soybean production merit investigation to fully understand the implications of soy consumption on human health and disease. This holistic perspective is in line with the growing interest in addressing food security challenges while promoting individual wellbeing (10).

The manuscripts featured in this Research Topic collectively illustrate the complex relationship between soy consumption and human health. From metabolic syndrome and cancer risk to food security, these contributions provide a holistic perspective on the role of soy in shaping our wellbeing. As the field continues to evolve, we expect that these findings will contribute to evidence-based dietary recommendations and strategies for promoting health and preventing disease. However, in order to make further progress in this field, interdisciplinary collaborations between nutritional researchers and environmental scientists are essential. Exploring the connections between soy's nutritional value, potential health benefits and environmental implications can lead to comprehensive knowledge that informs dietary guidelines, health interventions and sustainable practices. By embracing this integrative approach, researchers can lead the way to a more sustainable and health-conscious future, in which the link between soy, human health, and the environment is thoroughly understood and effectively disseminated to both practitioners and the general public.

## Author contributions

ML: Writing—original draft, Writing—review and editing. AF: Writing—review and editing. GR: Writing—review and editing.
